# Examining the Role of the Health Belief Model Framework in Achieving Diversity and Equity in Organ Donation Among South Asians in the United Kingdom

**DOI:** 10.3389/ti.2023.11243

**Published:** 2023-07-17

**Authors:** Agimol Pradeep, Titus Augustine, Gurch Randhawa, Paula Ormandy

**Affiliations:** ^1^ NHS Blood and Transplant, Watford, United Kingdom; ^2^ Manchester Centre for Transplantation, Manchester Royal Infirmary, Manchester University NHS Foundation Trust, Manchester, United Kingdom; ^3^ University of Bedfordshire, Luton, United Kingdom; ^4^ School of Health and Society, University of Salford, Salford, United Kingdom

**Keywords:** education, knowledge, South Asian, organ donation and transplantation, health belief model framework

## Abstract

Organ donation continues to be low among ethnic minorities in the United Kingdom (UK), especially within the South Asian community, with a disproportionate number of patients of South Asian ethnicity awaiting organ transplants. In 2020/21, Minority Ethnic (ME) patients comprised almost a third of the national transplant waiting list, highlighting the continued imbalance between the need for transplants in South Asian communities and the availability of suitable organs. Median waiting times for transplants show that, generally, white patients wait less time than ME patients; Only 39.5% of ME families consented to proceed with deceased organ donation when approached compared to 69% of white families. How to increase awareness among the South Asian community on the scarcity of organ donors continues to be a growing challenge facing the healthcare system in the UK and globally. This article reflects on the education strategy implemented using the Health Belief Model. It provides a detailed framework with which to consider the rationale that led to a specific behaviour, in this case organ donation among the three major ethnicities (i.e., Indian, Pakistani, Bangladeshi) within the South Asian community as part of a single study.

## Graphical Abstract

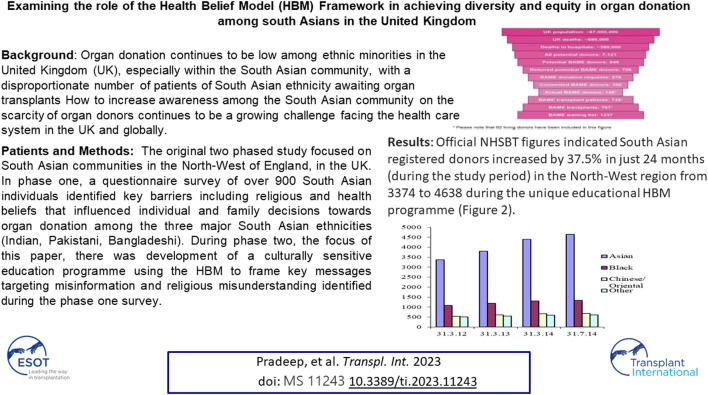

## Introduction

**FIGURE 1:**
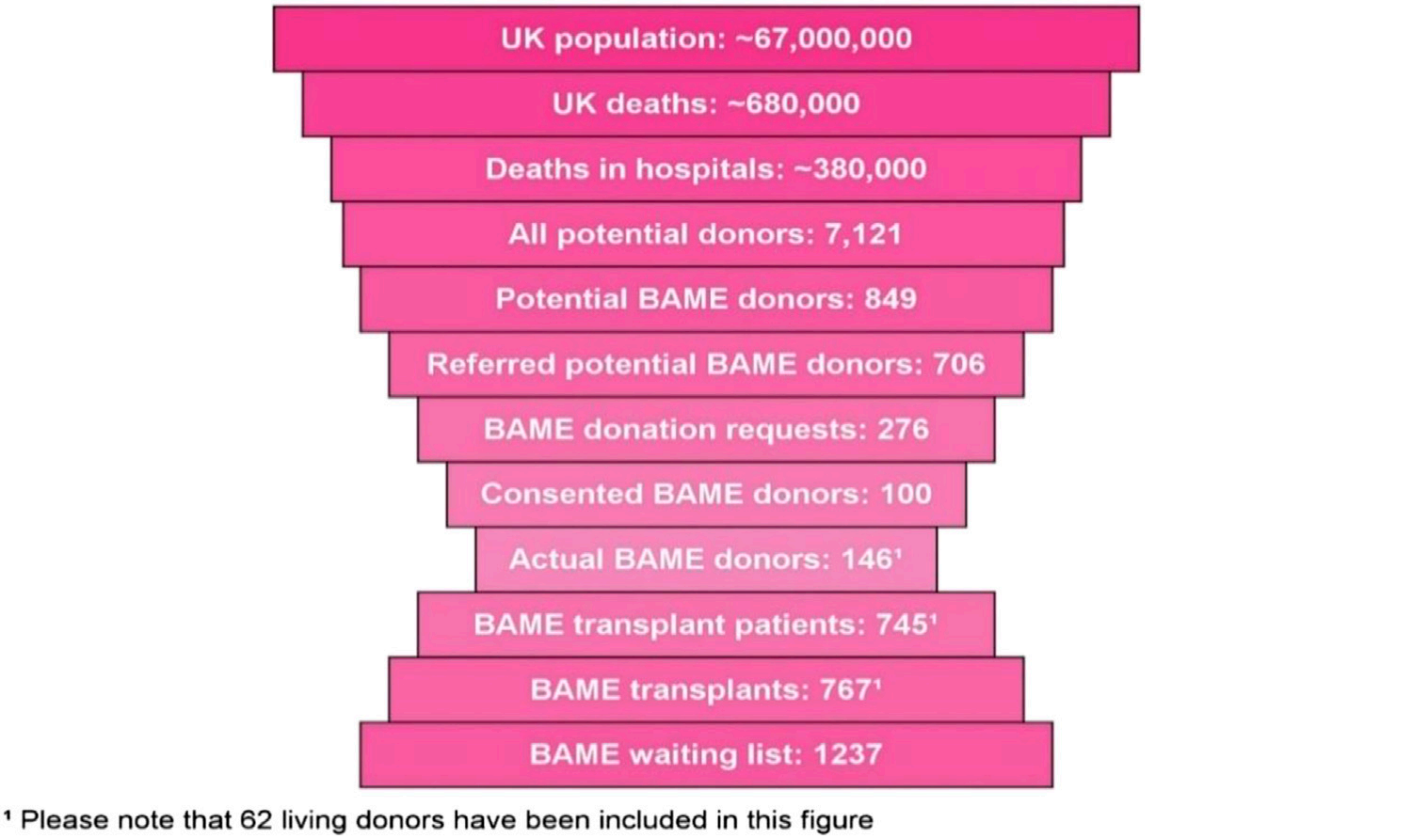
UK BAME potential organ donor population (April 2020–March 2021) Source: [2].

Organ donation continues to be low among ethnic minorities in the United Kingdom (UK), especially within the South Asian (representing individuals from India, Pakistan, and Bangladesh) community, with a disproportionate number of South Asian patients waiting for transplants, because suitable matches are more often found between individuals of the same ethnic group [1, 2]. Minority Ethnic (ME) patients represent almost a third of those waiting for a lifesaving organ transplant [2]. Median waiting times to transplant in the UK show that, generally white patients wait less time than ME patients. For kidney transplants, ME patients wait almost a year longer than white patients (median waits are 824 days for black, 682 days for Asian, 678 days for other ME and 527 days for white people). Donors of Asian ethnicity (2020/21) represented only 3% of deceased kidney donors and comprised 16% of recipients of deceased donor’s kidney transplants, however, make up 19% of the transplant waiting list in the United Kingdom (Figure 1). During the same period only 39.5% of ME families agreed to consent to proceed with deceased organ donation when approached compared to 69% of white families. Reasons reported for declining consent to donation by ME families includes difficulties because organ donation was not something discussed with their deceased relative and concerns regarding alignment of organ donation with their religious beliefs [2].

Even though National Health Service Blood and Transplant (NHSBT) and Department of Health (DH) identified the scarcity of South Asian donors two decades ago, it was only relatively recently that a sustained education campaign to address this was deployed [3]. The Community Investment Scheme (CIS) was funded nationally and led by the community itself at a local level in the last 4 years. There remains limited evidence through research studies on what campaigns or interventions work within the South Asian communities, why, and how [4].

This paper supports the notion of a whole community approach and provides a framework for culturally sensitive education using the Health Belief Model (HBM) [5], alongside identifying key South Asian community influencers to improve the equity and diversity of ME organ donation.

### Patients and Methods

The original two phased study [6] focused on South Asian communities in the North-West of England, in the United Kingdom. In phase one, a questionnaire survey of over 900 South Asian individuals identified key barriers including religious and health beliefs that influenced individual and family decisions towards organ donation among the three major South Asian ethnicities (Indian, Pakistani, Bangladeshi) [7].

During phase two, which was the focus of this paper, a culturally sensitive education programme was developed using the HBM to frame key messages targeting misinformation and religious misunderstanding identified during the phase one survey. The HBM is a psychological model to explain and predict health behaviours by focusing on the attitudes and beliefs of individuals. It has been applied widely in different health contexts [5]. The model focuses on the cost and benefit, which the individual perceives to be inherent in the specified organ donor behaviour. More importantly it evaluates how susceptible an individual may feel from the organ donation behaviour, the benefits from being a donor, the barriers stopping them donating an organ or any internal or external cues that influence them to be a potential organ donor [5, 6].

### Core Educational Content Included

• Perceived severity and susceptibility—plight of South Asian community, threat/prevalence of Chronic Kidney Disease (CKD), scarcity of organs, transplant waiting time, improved match within same ethnicity
• Perceived barriers—religious clarification that donation is acceptable, myths around organ donation process including: respect in handling the donor body, burial rituals, disfigurement
• Perceived benefits—helping South Asian people, gift of life, way of serving God, real stories

Organ donation and transplant education information was delivered by the South Asian educators, at over 289 community events, over 24 months which included: 127 Religious events (23 Muslim, 24 Hindu, 8 Sikh, 12 Jain, 1 Nepal, 56 Christian, and 3 multi-faith), 134 social community events, 24 health outreach events, and four university events targeting students and staff. Perceived severity and susceptibility awareness was raised through “real life” South Asian patient stories, sharing the experiences/struggles of those individuals with CKD and the positive experience of post-transplant patients, through social and digital media to influence the perceptions and opinions of the South Asian families and the community towards organ donation. Different education strategies were applied to the different meetings to engage first community influencers/leaders, then individuals and the wider community.

## Results

**FIGURE 2:**
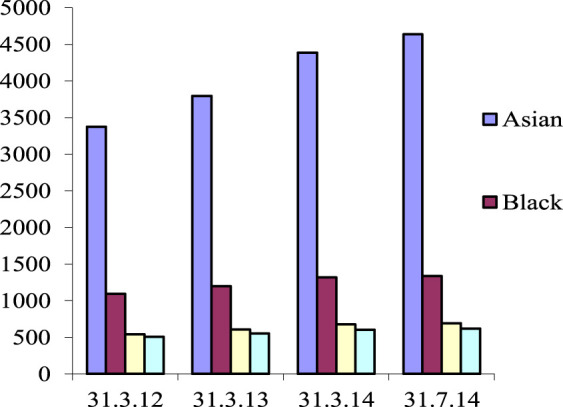
National Organ Donor Registry Ethnicity Data from UK Northwest during the study period. Source: [6].

Official NHSBT figures indicated that South Asian registered donors increased by 37.5% in just 24 months in the North-West region from 3,374 to 4,638 during the unique educational HBM programme (Figure 2). In reality the sign up of South Asian organ donors totalled 2,874 people across the different peer education sessions. Delays and issues with coding organ donor forms to track registrants to the project resulted in a loss of 522 coded forms identified after data reconciliation, and a failure to track the first 1,088 South Asian organ donor registrants for the first 6 months of the project.

The annual number of Asian deceased donor organs donated throughout the North-West ITU sites was low prior to the project but was observed to increase over the study period. In 2011–2012 eight Asian families were approached for organ donation without consent from any family. Eleven families were approached in 2012–2013, and one family provided consent. After intensive community and family education in 2013/2014, eight potential Asian donor families were approached, and three families consented to organ donation. This reinforced the need to continue to educate families, particularly if they were not themselves registered organ donors.

The findings demonstrated that using an HBM targeted education programme directly increased South Asian organ donor registrations. It highlighted the fact that increasing the individual or collective understanding that South Asians as a community were susceptible to kidney disease, that organ donation does not impede religious beliefs, and changing people’s health beliefs confer benefit for the community through organ donation was a successful educational strategy. What was important to note was that many individuals within the ME group claimed to have “*never heard these targeted messages before*” which suggested that targeting perceptions in susceptibility and the severity of the illness, and prevalence, such as “*this could happen to you*” education was effective to foster community attention (Table 2). These key elements (Box 1 and Table 1) within the HBM education programme influenced organ donor registrations. To address sensitive health issues, educators need an awareness of cultural differences and how these differences affect their health. Understanding needs of culturally directed health education by South Asian peers increased the number of registered donors over a short period of time. A pre-meeting with respected gate keepers (religious and community leaders) across different communities gained their trust and support for the education programme, increased access to the community, encouraged listening, and fostered community attention (Table 1, examples of key learning). South Asian individuals with a lived experience story within the community reinforced the accuracy of the information delivered and for some, directly influenced a change in health beliefs. Face to face education sessions were more successful in encouraging individuals to sign up to the organ donor register, demonstrating the importance of trust in the person delivering the message.

**TABLE 1:** Key influencers and education strategies using HBM.

Event, experience and activity—Field notes	Key rules of engagement
Meeting A	Pre-event meeting with editorial team
• Pre-meeting with event organisers, educated about scarcity of organ donation, educated on key susceptibility and severity messages, Committee members shocked as this was the first time, they had heard this information, they wanted to do their best to support the issue	Identify key information to generate passion for cause
• Wrote article about the organ donation shortage in the native language and provided small video reinforcing the key messages of severity and susceptibility	Publish information to increase awareness prior to an event
• Published article 3 days prior to the meeting, prepared the audience, informed readers they can join the ODR during the meeting in presence of expert for clarification	Influencer—South Asian Press
• 182 individuals registered from an event attended by 400	
Meeting B	Pre-meeting with community leader
• Meeting community leader in advance, before the event, educated on key susceptibility and severity messages	Educate key influential people to gain support for cause
• Community leader keen to promote the key message	Pre-event advertising via social media by the influential community leader
• Advertised the facility of ODR registration along with his own special request for people to join the register via social media and advertising material	
• 134 individuals registered from an event attended by 200, people more willing and already informed	Influencer—Community Leader
Meeting C	Pre-meeting with religious leader
• Meeting with religious leader and discussion surrounding key susceptibility and severity messages	Educate then gain support for cause and permission to access group
• Gained access and permission to have 30 min during one of their religious group session	Endorsed by South Asian Scholar in respected position
• Religious leader introduced the researcher to the group and his positive view on organ donation	Religious Leader available to support the education with positive religious interpretation
• Religious leader willing to clarify religious concerns during education session –more convincing than health professional	
• 15–20 min presentation on the topic by the researcher and 15 min question and answers, led by the religious leader	Influencer—Religious Leader
• 18 individuals registered from an event attended by 26	
Meeting D	Pre-meeting with local organisers to confirm purpose
• Researcher introduced to event organisers by Transplant patient, influential in local association	Recommended by respected member of community
• Group already aware of topic and impact of CKD from the patient belonging to their community	Use of “real” life experience—inside group supporter
• Local champion (transplant patient) enabled access to future events and endorsed education	Influencer—person with lived experience from within community
• 46 individuals registered from an event attended by 70	

## HBM Education Increased Organ Donors and Improved Equity—Discussion

Fundamental to the HBM is that a person’s belief of a personal threat combined with their belief in the effectiveness of the proposed behaviour predicts the likelihood of that behaviour [5]. Health beliefs are affected by numerous factors when applied to the topic of organ donation; this included underlying knowledge, attitudes, religion, ethnicity, community influences, misconceptions, mistrust, and misinterpretation. People are rational in their thoughts and actions and take the best health supporting action if they feel that it is possible to address a negative health issue, believing in taking the proposed action to expect a positive result [5, 8]. Six key concepts serve as the foundation for the HBM: perceived susceptibility, perceived severity, perceived benefits, perceived barriers, cues to action, and self-efficacy [5]. These concepts are discussed below in turn with specific reference to the South Asian community’s collective perceptions and individual behaviour; being an organ donor, consenting to organ donation, or potentially needing an organ.

Box 1 **Perceived severity and susceptibility—Community education key messages**

• In the United Kingdom, three people die every day waiting for an organ (more than 1,000 per year)
  • Nearly 5,000 people die each year in circumstances where their organs could be donated, and less than 1,500 go on to donate
  • Better match and outcome if the donor and recipient are from the same ethnicity
  • South Asians are more prone to develop CKD
  • Blood group and tissue type match
  • More than 10,000 patients on the waiting list in total and 17% are South Asians

### Perceived Susceptibility

Perceived susceptibility is the assessment of the likelihood of an individual (or the collective community) developing a specific condition [5]. South Asians living in the UK are three to four times more likely to need a kidney transplant compared to the white population, reflecting higher inherent rates of diabetes and hypertension, both of which are major causes of CKD [9]. There are a disproportionately high number of Asian patients on the kidney transplant waiting list. The most successful matched donors come from the same ethnic community [1]. However, donors from the South Asian community were not forthcoming, coupled with a lack of knowledge and awareness of the needs of the South Asian community [1, 3]. This crucial information was employed within the education model to really expose and increase a person’s knowledge of their individual and community perceived susceptibility.

Within this study we found that, when there was improved understanding of the perceived susceptibility of developing CKD themselves, or in a family member, there was recognition of the positive outcome of organ donation, which directly influenced organ donor registrations among the South Asian community. Many individuals registering as organ donors expressed the view that this was the first time they had heard this message, despite countless campaigns over the last two decades [6]. Indeed, feedback from some participants indicated that as a result of knowing that their organs when donated would more than likely allocated to someone of the same ethnicity, a “*gift of life*” motivated their behaviour to donate or influenced their decision to consent to their family members organs being donated. The South Asian community had strong bonds of the specific community related benefits in organ donation, which were taken on board. Awareness and motivation were important aspects of the education model to promote the organ donation message among the South Asian community [10].

### Perceived Severity

Perceived severity was defined as how severely a person (or collective community) may be affected if they were to suffer the condition [5]. Like susceptibility, the severity of the condition and the impact on individuals in that community was evident in the lack of organs being donated, and the increased time of an ethnic South Asian’s wait for a kidney transplant [11]. Severity is increased through the difficulty in blood groups and tissue type matching, to offer the best possible kidney match [2]. Ultimately, the gold standard treatment option of transplantation is significantly reduced due to the lack of donors from the same ethnicity group [12]. These facts once understood were shocking for some, especially for those with relatives with renal failure, and stimulated the motivation behind the behaviour change to register as an organ donor. For individuals and families unaffected by kidney disease, their perceived severity may remain low. However, to further stimulate perceived severity we co-opted the support of those (Table 1) who know that they themselves or someone they know may need a transplant at some point in time, further stimulating the collective organ donor registration.

### Perceived Benefit

Perceived benefit is the belief in how effective the action taken will be in mitigating the problems of the condition [5]. In this case the perceived benefit of organ donation being: the increase in available South Asian donor’s will translate into opportunities for ME patients with organ failure to receive the optimum and gold standard treatment option of transplantation, and thereby improving their quality of life [12]. The perception of this benefit was reinforced by their religious or community leader (a key community influencer), who interpreted the positive benefit from the different spiritual and community perspectives. For example, benefit gained from a selfless act provides reward in the afterlife (Buddhism, Sikh, Hindu, and Islam); organ donation deemed a “*gift of life*” without any personal gain, an act satisfying divine perceptions. Individuals often join the organ donor register because they want to be a hero by saving or improving the lives of others [7, 13]. Impactful education transpired into promoting and explaining the notion that, by donating deceased organs, up to nine people could benefit following their death influenced some to take the decision to donate. Morgan et al. (2006) discussed the notion that using strong community bonding to educate on the subject of tissue type and matching organs within ME communities was more beneficial and successful, which motivated people to sign up as an organ donor to benefit their own community.

### Perceived Barriers

Perceived barriers are the individual’s perceptions of the difficulties one would encounter in taking the proposed actions, including both physical and psychological barriers [5]. Barriers leading to the shortage of Asian organ donors joining the organ donor register included: medical mistrust, religious beliefs, mistrust in the healthcare system, lack of awareness, misinterpretation of faith, and lack of discussion by the health professionals [7]. A lack of motivation to register as an organ donor was influenced by uncertainty about the donation decision, a lack of knowledge about the process of organ donation, and a simple lack of knowledge about how and where to register [7, 14]. The HBM perceived barriers informed the education content. A comment from a participant suggested that “*feeling blamed for not registering would not have been helpful*,” rather the non judgemental delivery of the education enabled people to listen with a positive attitude and motivated some to join the register.

### Cues to Action

Cues to action are the strategies or prompts that allow a person to feel that they are ready to take the prescribed action [5]. Research shows that various media such as newspapers [15], television dramas [16] and television news [17], can serve as prompts for individuals and strategies to activate willingness to be an organ donor. The Asian community needs to be informed and reminded of the transplant crisis it faces and their shared responsibility to contribute donated organs. A number of strategies or “Cues to actions” were used to stimulate increased organ donation registrants within the HBM education.

Increasing public awareness of the National organ donor register as a means to record preferences on this issue is clearly a worthwhile goal [18]. Educational programmes by primary healthcare professionals about organ donation and transplantation could directly influence the attitudes and knowledge of potential donors [19]. A local research study identified that the South Asian community would trust their General Practitioner (GP) asking them to donate, and this would positively influence their decision [20].

Targeting minority ethnic press and media was an effective way to deliver information to people in this study (Table 1); Modern media shapes not only what people think about but also how they think about issues [21]. Culturally directed health education (by South Asian educators) increased the number of registered donors over a short time [20]. Similarly, peer education within ME groups has been shown to be effective, particularly concerning disease prevention [22].

In the UK in the last 5 years (data from 1 April 2017–31 March 2022), despite opt-out implementation, around 60% of ethnic minority eligible donors’ consent/authorisation was declined by their families, to proceed with organ donation [2]. A lack of discussion by healthcare professionals with families of the potential donor has been suggested as a reason for lower donation rates among Black families in the United Kingdom [19, 23]. Misconceptions about organ donation can be improved through community and family education and awareness [24] which in turn can increase the number of new donors [25]. However, organ donation consent by the family member depends on the skill of transplant/donor coordinators influencing a relative’s decisions to offer organ donation [26]. Van Embden et al*.* (2008) advocate that whatever the approach, prompt or cue for action, an essential component is the involvement of the whole team of healthcare workers, sensitive to the values and the traditions of ethnic communities.

This study [6] demonstrated that a whole systems approach [4] to donor and transplant education using the HBM to frame key messages, delivered by educators from the South Asian community who themselves were aware of the cultural perceived barriers, supported by community influencers, improved the uptake and action of the community to register as organ donors. This is reinforced by previous studies identifying that education alone is not sufficient motivation [1]. Taking time to identify the right educator is the key to influencing the views of a community. Additional findings can be drawn from the HBM education programme to inform education models of the future. For example, to address sensitive health issues, educators need to be aware about the cultural differences and how these differences affect their health and understanding needs. Culturally directed health education by South Asian peers increased the number of registered donors over a short time period [20]. Targeting respected gate keepers to communities, gaining their trust, and bringing them on board with the education programme opens doors and gathers the attention of the community to encourage them to listen. Real South Asian people with a story to tell influenced misinformation and changed health beliefs.

Trust in the South Asian educators was key to delivering the message and overcoming mistrust at the outset. A Hindu religious leader commented: *“in the past we have been approached by a health worker not from an Asian background with a request to have an opportunity to speak to the congregation about organ donation, but as I was not sure about the intentions and the rationale for his approach I decline permission. I now understand and I will definitely support your campaign.”* Like other studies, educators from the same community were not sufficient, face-to-face delivery was also important; so the community could decide if they trusted the educator, before they trusted the message [11].

### Self-Efficacy

Self-efficacy was not measured within this study but in the HBM it is described as confidence in one’s ability to act and this can be increased through information, knowledge, encouragement, and support [5]. Within this context self-confidence of an individual could influence their belief in the “*gift of life*,” influenced by the perceptions of the community, the religious leader or family. To overcome this, we targeted and educated the community as a whole, to drive peer support and community belief in the messages, which in turn potentially influenced the confidence of an individual, but this requires further research.

Drawing together the key learning (Table 2) from the HBM study as a whole, a number of strategies were influential in the success of the education campaign which considerably increased the number of organ donors to be registered. Using the key learning to build national campaigns in the future stimulating whole community ownership and action to address the lack of ME organs will improve the equity and diversity of available organs for the future.

**TABLE 2:** Overview of key HBM concepts and organ donation.

	Organ donation	Possible cues for action
Perceived susceptibility	• Increase awareness of:	✓ Provide accurate information to increase knowledge
	- CKD prevalence	✓ Information on how to register on ODR
	- scarcity of ethnic organs	✓ Personal stories and experiences from real people to raise awareness
	- time on waiting list	✓ Use of South Asian Media/TV
	- blood and tissue typing	✓ Reassurance of best possible treatment by healthcare professionals when dying
	- overall plight of South Asian community	
Perceived severity	• Identify level of risks to self, family and wider community	✓ GP recruitment or provision of information for ODR
	• Concerns over deceased organ donation, less active treatment by medical staff to save own life	✓ Reinforce message that donation will directly benefit ethnic minority community
Perceived benefits	• Increased number of available ethnic minority organs incase needed by individual, family or member of wider community	✓ Share and disseminate Fatwa advice to wider community—clarify religious stance for different groups
	• Reduced time on the transplant waiting list for South Asians	✓ Engage local religious leaders to spread positive message, encourage wider religious debate
	• Increased number of South Asians receiving the optimum treatment option of transplantation reducing the number on dialysis	✓ Cultural reassurance as to how a dead body is managed when donating an organ
	• “Gift of life” selfless act to help others fulfilling religious and cultural practices—feeling of being a “hero”	✓ Educate families and in particular elders
Perceived barriers	• Lack of knowledge and awareness of need and how to become ODR	✓ Peer education or education by a person who understands and belongs to the South Asian community
	• Religious misinterpretation	
	• Religious leaders and family elders	✓ Sustained education programs (maybe earlier in schools/universities)
	• Poorly trained health professionals not culturally sensitive	
	• Mistrust in health system to sustain life of ill person	✓ Training/education of whole team of health professionals on South Asian culture and religion to ensure effective communication and trust
	• Inappropriate cultural management of deceased donor	
Self-efficacy	Encourage individual confidence in own decision making, confidence to make appropriate decisions for next of kin, and wider community	

## Key Points of Learning

• The HBM is successful in framing key messages that will impact on the South Asian community to raise awareness and stimulate action to register as an organ donor or allow the donation of family organs to help the wider community.
• Perceived susceptibility, severity, and benefit are useful to frame key messages that will foster collective and individual action across ME communities. The belief that they are doing something that will benefit the wider community is a cue to action.
• Drawing on real life stories from people within the community provides authenticity to the need for organ donors and emphasises the reality of the problem for people, supporting the HBM key messages as to the benefit of matched ME organs.
• Employing skilled peer educators from the same community, aware of the cultural barriers to organ donation, increased the likelihood of community access, the message being heard, and the educator being trusted.
• Gaining the support of key influencers (such as religious and community leaders, media editors, local figures) within a community will improve access to community events, endorse the importance and trust in the key messages, and positively influence the community to take collective action.
• Religious leader support to help explain and discuss barriers within the community allays fears created by misinformation and myths, which influences support for organ donation in ME groups.
• Targeting minority ethnic press and media is a potentially effective way to get information to people within a community if the media is a trusted source of information within the community.

## Conclusion

A South Asian education programme based on HBM theory successfully increased the diversity of the organ donor register base, showing promise of a way to begin to reduce health inequalities for people on the transplant waiting list, a long-term national NHSBT vision for 2030 [27]. Perceived susceptibility, severity, barriers, and benefits of organ donation need to be communicated effectively to foster organ donation and family support for the “gift of life” message. Following the HBM framework provided guidance to the educator, to understand the South Asian individual’s beliefs, knowledge, attitudes, and behaviours as determinants of willingness to become an organ donor. A combination of multi-level strategies that target the whole ME community; HBM education programmes, using community educators, and community influencers are required to instigate action. This whole systems approach [4] at a local, national, and international level can tackle the scarcity of ME donors linked to time served initiatives and start to seriously improve the number of ME donors coming forward in the UK similar to other European countries [28].

## Data Availability

The original contributions presented in the study are included in the article/supplementary material, further inquiries can be directed to the corresponding author.
